# Longitudinal associations between play experiences and trajectories of preschoolers' mental health from April–July, 2020

**DOI:** 10.1002/jcv2.70076

**Published:** 2026-02-10

**Authors:** Helen F. Dodd, Ella Patterson, Simona Skripkauskaite, Peter J. Lawrence

**Affiliations:** ^1^ Children and Young People's Mental Health Research Collaboration (ChYMe) Exeter Medical School University of Exeter Exeter UK; ^2^ Oxford Health NHS Trust Oxford UK; ^3^ Department of Psychiatry University of Oxford Oxford UK; ^4^ Department of Experimental Psychology University of Oxford Oxford UK; ^5^ School of Psychology University of Southampton Southampton UK

**Keywords:** contact with nature, COVID‐19, mental health, physical activity, play, preschoolers, United Kingdom

## Abstract

**Background:**

Play provides an important foundation for a healthy childhood but longitudinal data exploring how play experiences relate to children's mental health over time is scarce. In this study, data on preschool‐aged children's activities and mental health during Covid‐19 related restrictions was used to explore how *where (inside/outside)* and *with whom* children played related to internalising and externalising problems over time.

**Methods:**

UK based parents/carers (*n* = 1028) of preschool‐aged children (2–5 years) completed an online survey at four time points between April and July 2020. The survey asked how much time in the previous week their child had spent: Playing inside; Playing outside; Playing alone; Playing with a parent; Playing with another child in their household; and their child's mental health (Strengths and Difficulties Questionnaire internalising and externalising scales). Four hierarchical linear regression analyses were conducted to examine associations between *where* children play (inside/outside) and (1) internalising and (2) externalising symptoms, and *with whom* children play (with parent, other child, alone) and (3) internalising and (4) externalising symptoms. Effects of linear and quadratic time, and interactions between play and time were examined. Parent mental health, parent education, contact with nature and physical activity were included as covariates.

**Results:**

Both inside and outside play was associated with less severe internalising problems (beta = −0.23 [SE = 0.10]; beta −0.54 [SE = 0.19]) and, in statistical interaction with time, less time playing inside was associated with a stronger improvement in externalising problems over time (beta = 0.77 [SE = 0.23]). Further, more time playing with other children was associated with less severe internalising problems (beta = −0.50 [SE = 0.13]) whereas playing alone was associated with more severe internalising problems (beta = 0.28 [SE = 0.10]).

**Conclusion:**

Varied play opportunities are related to young children's mental health. Even during a pandemic response, children should be given opportunity to play with other children and play outdoors wherever possible.

## Key Points

**What's known?**

- Cross‐sectional research suggests there is a relationship between play and mental health in children.
- It is unclear how the specific context of children's play affects associations with mental health.

**What's new?**

- For internalising problems, inside and outside play, and more time playing with other children were associated with fewer symptoms. More time playing alone was associated with more symptoms.
- For externalising problems, no main effects of play were found but symptom levels remained relatively stable over time for children who spent more time playing inside, whereas children who spent less time playing inside had a decreasing symptom trajectory over time. Time spent playing with parents was not significantly associated with preschoolers' mental health.

**What's relevant?**

- Understanding how children's play activities can be used to improve their mental health during periods of enforced isolation is essential to inform the development of future policies and guidance.

## Graphical abstract

Based on data collected monthly from April to July 2020, we found that time spent playing outside and with other children were associated with lower internalising problems across time in preschool‐aged children, whereas more time spent playing alone was associated with more severe internalising problems. These associations held after controlling for activity levels and contact with nature.

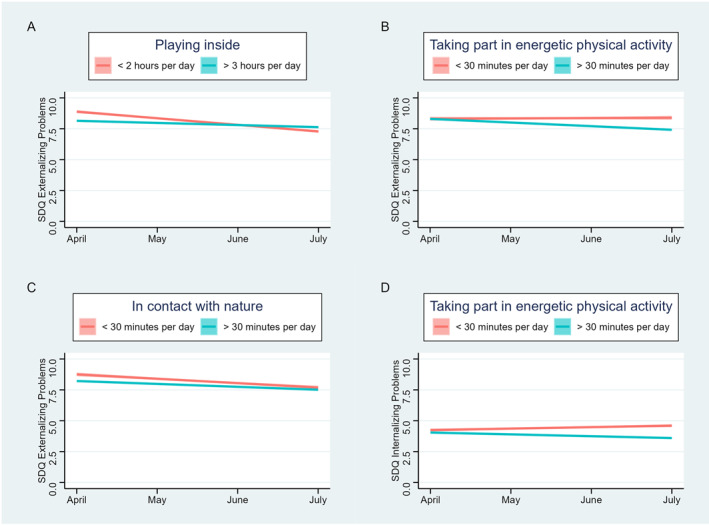

## INTRODUCTION

Play has been defined as ‘any behaviour, activity or process initiated, controlled and structured by children themselves … non‐compulsory, driven by intrinsic motivation and undertaken for its own sake, rather than as a means to an end’ (United Nations, 2013). The broad role of play in supporting children's healthy development has long been recognised by developmental psychologists (Piaget, 1962; Rubin, 1982; Vygotsky, 1978). Play is proposed to support healthy development across a range of developmental domains including social development, emotion regulation and cognitive development (Andersen et al., 2023; Barnett, 1990; Singer et al., 2006; Zhao & Gibson, 2022). There is also emerging evidence that more time spent playing may be linked to better mental health in children (e.g., Dodd et al., 2023; Zhao & Gibson, 2023) but there is a lack of longitudinal research examining this association and it is currently unclear whether play in specific contexts (e.g., indoor vs. outdoor play, play with other children vs. solitary play) impacts associations with mental health. Due to the restrictions to children's activities during the Covid‐19 pandemic, data collected over this period provides a unique opportunity to examine how specific play experiences relate to children's mental health over time.

Throughout the COVID‐19 pandemic children's mental health was a particular public health concern. In England, rates of probable mental disorders in children aged 7‐ to 16‐year increased from 12.1% in 2017 to 16.7% in 2020 (NHS Digital 2022). Although there has been less focus on younger children, preschoolers' mental health was also negatively affected by the pandemic (Cantiani et al., 2021; Ding et al., 2022). For example, in our study of UK preschoolers' mental health symptom trajectories from April 2020 to March 2021 we found a general pattern of symptom improvement at the end of the first UK lockdown, followed by worsening of mental health symptoms during subsequent national lockdowns (Lawrence et al., 2023). These findings suggest that the restrictions had a negative impact, on average, on preschool children's mental health. Nevertheless, there was significant heterogeneity. Whilst some of this variation was due to sociodemographic factors, such as household income, and psychological factors such as parental distress (Guzman Holst et al., 2023; Frigerio et al., 2023), other aspects of children's experiences, such as opportunities for play in different contexts may also have affected their mental health during this period.

Social play with other children reduces feelings of social isolation and preschooler peer play is associated with better mental health in middle childhood (Zhao & Gibson, 2023). During the pandemic, preschoolers' opportunity for social play and social interactions were significantly reduced (Graber et al., 2021). In the UK, childcare settings were closed to most children, public playgrounds were closed, and there were restrictions regarding the amount of time children were allowed outside and whom they were allowed to see, which prevented contact with children outside the family. There is some indication that, when children were able to play with other children, they had better mental health during Covid‐19 related restrictions. For example, in Italy, among 2‐6‐year‐olds, contact with kindergarten mitigated the severity of mental health symptom deterioration during the early stages of national lockdown (Cantiani et al., 2021). Similarly, UK preschoolers who attended childcare showed greater improvements in symptom severity over time than preschoolers who did not attend childcare (Lawrence et al., 2023).

These findings do not provide clear evidence that play with others per se was driving these associations because attendance at childcare may affect preschool children's mental health in a range of ways. In fact, the presence of other children in the home was not associated with preschool children's mental health symptoms during the pandemic (after controlling for attendance at childcare), suggesting that play with other children may not drive these childcare effects (Lawrence et al., 2023). Alternatively, different social contexts of play might offer distinct benefits to children. For example: parent‐child play has been linked to good self‐regulation (Galyer & Evans, 2001) and closer parent‐child relationships (Kerns & Barth, 1995; Roy & Kumar, 2022); whereas playing alone is associated with increased creativity (Sumaroka & Bornstein, 2008) and accelerated language development (Zhao & Gibson, 2022); and social play is linked to emotion regulation (LaFreniere, 2011) and social integration (Barnett, 1990). To our knowledge no research has examined how specific social contexts of play are associated with children's mental health symptoms. Furthermore, there is a lack of longitudinal research tracking associations between play and mental health over time. For young children, social and emotional development is rapid, and shifts between healthy and unhealthy developmental trajectories can manifest within a short timeframe. Longitudinal data collected during the lockdown period of the Covid‐19 pandemic therefore provides an opportunity to address these gaps in the existing literature.

The location of children's play might also be an important factor in how play links with mental health. For example, more outdoor play, but not indoor play, in the year prior to the pandemic was associated with fewer internalising problems and more positive affect at the start of the restrictions (Dodd et al., 2023). To our knowledge, although outdoor play decreased and indoor play increased during the pandemic (Kourti et al., 2021), no research has examined how these changes in play over time were associated with changes in preschoolers' mental health. Where studies have examined outdoor play, they have primarily focused on children's physical activity, with reductions in outdoor play linked to less physical activity (Hyunshik et al., 2021). Given that physical activity is closely associated with mental health (Biddle & Asare, 2011; Rodriguez‐Ayllon et al., 2019), physical activity is an important potential confound of associations between play and mental health. Time spent in nature and green space is an additional confounding factor because a significant proportion of outdoor play happens in green spaces (Dodd et al., 2021) and there are strong links between contact with nature and mental health (Bell et al., 2019; Roberts et al., 2019; Tillmann et al., 2018).

In summary, play appears to be beneficial for children's mental health but there is a lack of longitudinal research and it is unclear whether the social context of play and location of play affect this association. The pandemic provided a context within which the potential impact of play for mental health in young children could be explored. In this study, we focus on preschool‐aged children and examine whether (a) who children play with, and (b) where they play, is related to changes in their mental health over time between April–July 2020 (to align with the first UK‐wide lockdown). We controlled for physical activity and contact with nature, along with relevant demographic characteristics. We hypothesised that more time spent playing outdoors and with other children would be associated with better mental health over time.

## METHODS

This study used data collected as part of the larger longitudinal study ‘Co‐SPYCE (COVID‐19: Supporting Parents and Young Children during Epidemics)’. The research protocol of the original study is available via the Open Science Framework (https://osf.io/rukpt/).

### Study design

The present study is a longitudinal, repeated measures, within‐subjects design using secondary data. Data from four monthly assessments (April–July 2020) are included. Full details of the measures are given in the sections that follow. The study has two continuous outcome variables: the ‘internalising problems’ and ‘externalising problems’ composites of the Strengths and Difficulties Questionnaire (SDQ; Goodman, 1997; Goodman et al., 1998). There are five exposure variables (playing inside, playing outside, playing alone, playing with a parent, playing with another child), and four covariates (physical activity, contact with nature, parent/carer educational attainment, and parent/carer mental health). Education was selected as a covariate to covary for socio‐economic status; education was preferable over household income due to missing data on the latter. Parent mental health, measured using the Depression, Anxiety, Stress Scale (DASS‐21), was included as it is an important predictor of child mental health and may also be associated with children's play opportunities (Lawrence et al., 2023).

### Participants

UK Parents/carers (over the age of 18 years) of preschool aged children aged between 2 and 4 years (in Scotland, up to 5 years) at the start of the study, were eligible to participate. Participants were recruited via social media, distribution through partner organisations, networks, charities and radio and television media. Across the four time points (April to July 2020) there were a total of 2529 participants (see Table 1). The current paper focuses on 1028 participants who completed at least two surveys (an initial survey and at least one additional monthly survey) between 17th April and 31st July, 2020. To be considered to have completed a monthly survey, participants must have completed the SDQ, the DASS‐21 and measures of children's activity that month. Additionally, to be included in the final sample, parents/carers must have reported their level of education attainment in their initial survey. Participant demographic information can be found in Table 1.

**TABLE 1:** Participant demographic information.

	April	May	June	July
	*n* = 454	*n* = 789	*n* = 713	*n* = 574
Parent gender				
Male	28	35	31	33
Female	426	753	682	540
Parent ethnicity				
Asian	9	27	23	19
White British	433	741	670	535
Any other*	7	13	12	14
Parent/carer education				
Completed GCSE/CSE/O‐levels or equivalent, completed post‐16 vocational course, No qualifications*	21	42	37	24
A‐levels or equivalent	47	56	52	48
Undergraduate degree	192	339	300	237
Postgraduate degree	194	352	324	265
Child mean age (SD)	2.94 (0.76)	2.97 (0.79)	3.01 (0.80)	3.11 (0.79)
Child gender				
Male	223	413	365	307
Female	231	375	347	266
Missing/other	0	1	0	1
Child ethnicity				
White British	420	712	640	512
Any other*	18	39	30	29
Child SEN				
Yes	17	29	23	20
No	437	760	690	554
Household income				
<£16,000 p.a.	17	32	26	20
>£16,000 p.a.	413	703	636	511
Prefer not to say				
Family composition				
Multiple adult household	430	738	669	541
Single adult household	24	50	42	33
Parent/carer mental health (DASS‐21)				
Mild (%)	365 (80.40)	658 (83.40)	594 (83.43)	482 (84.0)
Moderate/severe (%)	89 (19.60)	131 (16.60)	118 (16.57)	92 (16.0)

*Note*: For sub‐categories with a superscript*, some cells contained fewer than 5 participants. So, to prevent participant identification, we have aggregated such sub‐categories and reported these. For example, the ‘Parent ethnicity’ sub‐categories were originally ‘Asian’, Black’, ‘Middle Eastern’, Mixed race’, White’ and ‘Other ethnic group’. Some cells in the ‘Black’, ‘Mixed race’, Middle Eastern’ and ‘Other ethnic group’ sub‐categories had fewer than 5 participants, so we aggregated them to form ‘Any Other’.

Abbreviation: DASS‐21, Depression, Anxiety, Stress Scale.

### Procedure

Parents/carers provided informed consent and completed surveys online between 17th April and 31st July, 2020. For context, the UK‐wide lockdown was legally enforced from March 26th, 2020. A link to the follow‐up surveys was sent via email to each parent/carer one calendar month after they had completed their first survey and then each subsequent calendar month. Full procedural information is available at https://osf.io/rukpt/. Ethical approval for the study was granted by The University of Southampton Research Ethics Committee: ERGO 56217.

### Measures

#### Educational attainment

Parents/carers responded to a multiple‐choice question ‘What is your highest level of educational attainment?’. We created a binary variable, with 0, 1, 2 and 3 categorised as ‘up to and including further education’ (coded as one), and 4 and 5 categorised as ‘higher education’ (coded as zero).

#### Parent/carer's mental health

The DASS‐21 (Lovibond & Lovibond, 1995) is a self‐report measure designed to measure negative emotional states, with established validity in community samples (Henry & Crawford, 2005). The DASS‐21 comprises 21 items that correspond to 3 subscales, Anxiety, Stress and Depression. Subscales scores are combined to give a total score which ranges from 0 to 126. Participants' total DASS‐21 scores were grouped based on defined severity ranges (Lovibond & Lovibond, 1995). Group 0: Mild ‐ with total scores ≤40, indicating no difficulties or scores below the population mean, and group 1: Moderate/Severe with total scores ≥41, indicating moderate, severe and extremely severe difficulties (coded as one).

#### Children's mental health

Parents/carers reported on their preschoolers' mental health using the 25‐item preschool version of the SDQ (Goodman, 1997; Goodman et al., 1998). The SDQ was scored following the SDQ scoring guidance. Each item is scored on a three‐point Likert scale ranging from 0 (‘not at all’) to 2 (‘certainly true’). Items can be combined to create five subscales: ‘emotional symptoms’, ‘conduct problems’, ‘hyperactivity/inattention’, ‘peer relationship problems’ and ‘prosocial behaviour’. Item scores are summed to provide a total subscale score ranging from 0 to 10, with higher scores indicative of increased severity. An ‘internalising problems’ score is calculated by summing ‘emotional problems’ and ‘peer relationship problems’, and an ‘externalising problems’ score by summing ‘conduct problems’ and ‘hyperactivity/inattention’.

#### Children's activities (play, physical activity, contact with nature)

Seven separate measures of children's activities were used to define how much time in a week preschoolers had spent: Playing inside; Playing outside; Playing alone; Playing with a parent; Playing with another child in their household; Taking part in energetic physical activity (whether inside or outside); In contact with nature (plants, trees, grass, etc.).

Parents/carers reported on the following for each activity: ‘Over the last week, how much time per day did your child spend doing the following (on average)?’ with five possible answers‐ (0 = ‘Did not do’, 1 = ‘less than 30 min’, 2 = ‘30 min to 2 h’, 3 = ‘3–5 h’, 4 = ‘6+ hours’). The resulting ordinal variables were highly skewed, with very few children represented at the lowest and highest ends of the scale (see Supporting Information S1: Table S1). This made it difficult to model the ordinal structure reliably, and we therefore dichotomised the variables to retain statistical power and interpretability. Specifically, we created ‘low/high’ binary variables for each activity using a median split (‘low’ (0) = below the median, ‘high’ (1) = median or above). Specifically, for ‘Playing inside’, 0, 1 and 2 formed the ‘low’ category, and 3, and 4 formed the ‘high’ category. For ‘Playing with another child in the household’, 0 formed the ‘low’ category, and 1 to 4 formed the ‘high’ category. For all other activities, 0 and 1 formed the ‘low’ category, and 2 to 4 formed the ‘high’ category.

### Data analysis

Analyses were carried out using the lmer function within the lme4 package (v. 1.1‐2.3; Bates et al., 2015) of R Studio (version 4.0.5). All models were estimated using maximum likelihood estimation. We used hierarchical linear regression to compare models of children's mental health (dependent variable of either the SDQ internalising subscale score, or SDQ externalising subscale score) to examine the extent to which the outcome was accounted for by different components of preschoolers' play. This resulted in four examinations: (i) where children play and their internalising symptoms, (ii) where children play and their externalising symptoms, (iii) with whom children play and their internalising symptoms and, (iv) with whom children play and their externalising symptoms.

The analytical process for each examination consisted of a three‐step hierarchical regression approach. In the first step, we entered four covariates: linear and quadratic time (coded as 0 [April], 1 [May], 2 [June], 3 [July]), which capture the shape of symptom trajectory across the four timepoints, parent/carer educational attainment (as assessed at first survey completion) and parent/carer mental health. In the second step, we added the ‘where’ (inside and outside) or ‘with whom’ (alone, with parent/carer, and with another child) play variables as main effects, and their interactions with linear and quadratic time. In the third step, we added ‘physical activity’ and ‘contact with nature’ as main effects, and their interactions with linear and quadratic symptom change (time). A random intercept was included for each participant and the fixed effects were measures of children's activities and time. All variables in the model were time variant, meaning that they were measured at each timepoint, with the exception of parent/carer educational attainment. Taking this approach means that a significant main effect of a play variable indicates that the play variable is associated with the level of symptoms independent of time. In contrast, a significant interaction between a play variable and a time term indicates that the play variable is significantly associated with the shape of symptom trajectory over time.

To assess whether each block improved the modelling of preschoolers' mental health, we used a log‐likelihood change with *χ* ^2^ test significance of *p* < .05. Additionally, we examined Akaike Information Criteria (AIC) and Bayesian Information Criterion (BIC) likelihood comparisons. If the addition of a block improved the model, it should have a lower AIC, and preferably lower or similar BIC (Kuha, 2004), than the less constrained model (without the additional predictors). Furthermore, while our fit indices tell us which is the best of our models, they provide no coefficient of determination (*R* ^2^, in fixed effects models). Therefore, we followed Nakagawa and Schielzeth (2013) and reported their *R* ^2^ for mixed models, which comprises two values–the marginal *R* ^2^, which quantifies how much variance in the outcome is accounted for by fixed effects, or exposure variables, alone, and the conditional *R* ^2^, which quantifies how much variance is accounted for by the fixed and random effects (i.e., varying intercept between participants) together.

## RESULTS

### Where preschoolers played

#### Internalising problems

The results for the three hierarchical analysis steps are shown in full in Table 2. Across the steps, the models explained 70%–71% of variance in children's internalizing problems with 4%–5% of variance explained by fixed effects only (see Supporting Information S1: Table S2 for full model fit statistics). Overall, adding play variables and their interactions with time in Step 2 significantly improved the model fit from Step 1; Δ*χ* ^2^ (6) = 27.34, *p* < .001. Both play variables had significant negative main effects on preschoolers' internalising problem severity, ‘how much time children spent playing inside’ beta = −0.22 (SE = 0.10), *t* (2039) = −2.13, *p* = 0.034; and ‘how much time playing outside’ beta = −0.70 (SE = 0.17), *t* (2059) = −4.01, *p* < .001. Addition of physical activity and contact with nature variables in Step 3 further improved model fit; Δ*χ* ^2^ (6) = 15.55, *p* = 0.016. The negative main effects seen in Step 2 retained statistical significance (‘how much time children spent playing inside’ beta = −0.23 (SE = 0.10), *t* (2041) = −2.22, *p* = 0.027; and ‘how much time playing outside’ beta = −0.54 (SE = 0.19), *t* (1941) = −2.88, *p* = 0.004). Time spent in physical activity also had a negative main effect on children's internalising problems (beta = −0.42 (SE = 0.14), *t* (1993) = −3.09, *p* = 0.002). No interactions with symptom change over time were found.

**TABLE 2:** Regression coefficients (b) and standard errors (SE) for estimated effects per hierarchical modelling step for analyses examining where and with whom preschool children played as predictors of internalising problems.

	Step 1			Play where						Play with whom					
				Step 2			Step 3			Step 2			Step 3		
	*b*	SE	*p*	*b*	SE	*p*	*b*	SE	*p*	*b*	SE	*p*	*b*	SE	*p*
Time: Linear (T1)	**−0.21**	**0.08**	**0.006**	0.18	0.34	0.594	0.43	0.36	0.230	−0.05	0.40	0.891	0.47	0.44	0.288
Time: Quadratic (T2)	**−0.29**	**0.07**	**<0.001**	**−0.64**	**0.31**	**0.043**	−0.59	0.33	0.075	−0.19	0.35	0.582	−0.28	0.39	0.467
Parental education	**0.50**	**0.24**	**0.035**	**0.49**	**0.23**	**0.037**	**0.49**	**0.23**	**0.037**	**0.46**	**0.23**	**0.049**	0.45	0.23	0.052
DASS‐21	**1.29**	**0.14**	**<0.001**	**1.27**	**0.14**	**<0.001**	**1.25**	**0.14**	**<0.001**	**1.28**	**0.14**	**<0.001**	**1.26**	**0.14**	**<0.001**
Play inside				**−0.22**	**0.10**	**0.034**	**−0.23**	**0.10**	**0.027**						
*T1				0.01	0.19	0.942	0.05	0.19	0.798						
*T2				<0.01	0.18	0.985	0.02	0.18	0.888						
Play outside				**−0.70**	**0.17**	**<0.001**	**−0.54**	**0.19**	**0.004**						
*T1				−0.43	0.32	0.180	−0.03	0.38	0.937						
*T2				0.37	0.29	0.199	0.46	0.34	0.171						
Play alone										**0.28**	**0.10**	**0.005**	**0.28**	**0.10**	**0.006**
*T1										−0.03	0.17	0.852	−0.04	0.17	0.815
*T2										0.29	0.16	0.067	0.27	0.16	0.084
Play with parent										0.06	0.21	0.789	0.15	0.21	0.485
*T1										−0.11	0.38	0.768	−0.01	0.38	0.985
*T2										−0.37	0.33	0.263	−0.33	0.33	0.319
Play with child										**−0.51**	**0.13**	**<0.001**	**−0.50**	**0.13**	**<0.001**
*T1										−0.04	0.16	0.821	−0.03	0.16	0.850
*T2										0.10	0.14	0.496	0.11	0.14	0.432
Physical activity							**−0.42**	**0.14**	**0.002**				**−0.50**	**0.13**	**<0.001**
*T1							−0.50	0.27	0.060				**−0.54**	**0.25**	**0.032**
*T2							−0.22	0.24	0.361				−0.08	0.23	0.718
Contact with nature							0.03	0.13	0.811				−0.11	0.12	0.381
*T1							−0.24	0.24	0.318				−0.15	0.23	0.507
*T2							0.05	0.23	0.812				0.18	0.21	0.409

*Note*: Values in bold statistically significant at *p* < .05. T1 = linear time; T2 = quadratic time term. Step 1 was the same for ‘Play Where’ and ‘Play with Whom’ models. *T1 and *T2 indicate that the result is for the interaction with linear time and quadratic time respectively.

Abbreviation: DASS‐21, Depression, Anxiety, Stress Scale.

#### Externalising problems

**FIGURE 1:**
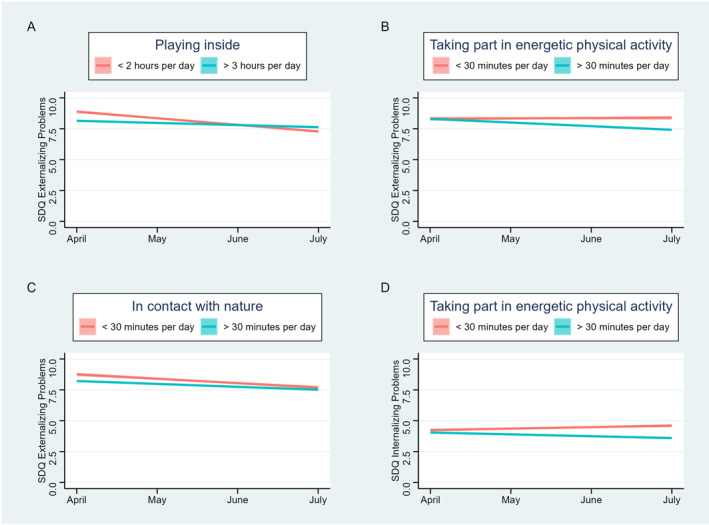
Predicted interaction effects. Figures depict associations between: (A) playing inside and externalizing problems over time; (B) physical activity and externalizing problems over time; (C) contact with nature and externalizing problems over time; and (D) physical activity and internalizing problems over time (note that although this interaction was found in the ‘with whom’ models and the ‘where’ models, the effect was almost identical so it is only shown once in this figure).

The results for all three steps are shown in full in Table 3. Across the steps, the models explained around 76% of variance in children's externalizing problems with 5% of variance explained by fixed effects only (see Supporting Information S1: Table S3 for full model fit statistics). Overall, adding play variables and their interactions with time in Step 2 significantly improved the model fit from Step 1; Δ*χ* ^2^ (6) = 17.49, *p* = 0.008. While neither play variable had significant main effects on preschoolers' externalising problem severity, the statistical interaction between linear time and how much time preschoolers spent playing inside, was significant; beta = 0.79 (SE = 0.23), *t* (1699) = 3.51, *p* < .001. Specifically, parents reported a decrease in externalising symptoms over time among preschoolers who spent less time playing inside, while externalising symptom levels stayed relatively stable in children who spent more time playing inside (Figure 1A).

**TABLE 3:** Regression coefficients (b) and standard errors (SE) for estimated effects per hierarchical modelling step for analyses examining where and with whom preschool children played as predictors of externalizing problems.

	Step 1			Play where						Play with whom					
				Step 2			Step 3			Step 2			Step 3		
	*b*	SE	*p*	*b*	SE	*p*	*b*	SE	*p*	*b*	SE	*p*	*b*	SE	*p*
Time: Linear (T1)	**−0.49**	**0.09**	**<0.001**	**−1.06**	**0.41**	**0.009**	**−0.87**	**0.42**	**0.040**	**−0.96**	**0.48**	**0.044**	−0.67	0.52	0.199
Time: Quadratic (T2)	**−0.39**	**0.08**	**<0.001**	−0.60	0.37	0.106	−0.60	0.39	0.124	−0.73	0.41	0.077	−0.90	0.46	0.050
Parental education	**1.17**	**0.31**	**<0.001**	**1.16**	**0.31**	**<0.001**	**1.15**	**0.31**	**<0.001**	**1.17**	**0.31**	**<0.001**	**1.15**	**0.31**	**<0.001**
DASS‐21	**1.62**	**0.17**	**<0.001**	**1.61**	**0.17**	**<0.001**	**1.62**	**0.17**	**<0.001**	**1.62**	**0.17**	**<0.001**	**1.63**	**0.17**	**<0.001**
Play inside				−0.15	0.13	0.237	−0.16	0.13	0.208						
*T1				**0.79**	**0.23**	**<0.001**	**0.77**	**0.23**	**<0.001**						
*T2				−0.15	0.21	0.490	−0.13	0.21	0.524						
Play outside				−0.31	0.21	0.127	−0.14	0.22	0.527						
*T1				−0.03	0.38	0.936	0.10	0.45	0.817						
*T2				0.35	0.35	0.307	0.16	0.40	0.689						
Play alone										−0.15	0.12	0.231	−0.13	0.12	0.293
*T1										**0.41**	**0.21**	**0.046**	0.36	0.21	0.081
*T2										−0.06	0.19	0.751	−0.06	0.19	0.764
Play with parent										−0.02	0.25	0.944	0.04	0.25	0.873
*T1										0.37	0.45	0.411	0.43	0.46	0.349
*T2										0.33	0.39	0.390	0.29	0.39	0.461
Play with child										−0.04	0.17	0.799	−0.04	0.17	0.828
*T1										−0.29	0.19	0.128	−0.30	0.19	0.111
*T2										0.14	0.17	0.389	0.12	0.17	0.476
Physical activity							−0.30	0.16	0.069				**−0.33**	**0.16**	**0.036**
*T1							**−0.84**	**0.32**	**0.008**				**−0.84**	**0.30**	**0.006**
*T2							0.11	0.29	0.708				0.13	0.28	0.646
Contact with nature							−0.10	0.15	0.503				−0.14	0.15	0.354
*T1							0.53	0.29	0.064				**0.54**	**0.27**	**0.048**
*T2							0.10	0.27	0.709				0.15	0.25	0.559

*Note*: Values in bold statistically significant at *p* < .05. T1 = linear time; T2 = quadratic time term. Step 1 was the same for ‘Play Where’ and ‘Play with Whom’ models. *T1 and *T2 indicate that the result is for the interaction with linear time and quadratic time respectively.

Step 3, with the addition of physical activity and contact with nature variables, was a significant improvement of model fit from Step 2; Δ*χ* ^2^ (6) = 14.24, *p* = 0.027. We found no significant main effects of play variables for preschoolers' externalising problems. The statistical interaction between linear time and playing inside was retained (Figure 1A); beta = 0.77 (SE = 0.23), *t* (1703) = 3.40, *p* = 0.001. Furthermore, there was a statistical interaction between linear time and physical activity; beta = −0.84 (SE = 0.32), *t* (1711) = −2.65, *p* = 0.008. Specifically, parents reported a decrease in externalising problems among preschoolers who spent more than 30 min per day being physically active, whilst for less active children symptoms were more stable over time (Figure 1B).

### With whom preschoolers played

#### Internalising problems

The results for all three hierarchical analysis steps are shown in full in Table 2. Across the steps, the models explained 70%–71% of variance in children's internalizing problems with 4%–6% of variance explained by fixed effects only (see Supporting Information S1: Table S2 for full model fit statistics). Overall, adding play variables and their interactions with time in Step 2 significantly improved the model fit from Step 1; Δ*χ* ^2^ (9) = 28.09, *p* < .001. Playing alone (beta = 0.28 (SE = 0.10), *t* (2231) = 2.79, *p* = 0.005) and playing with another child (beta = −0.51 (SE = 0.13), *t* (1978) = −3.82, *p* < .001) both had main effects on preschoolers' internalising problems. However, these effects differed in direction, such that more time playing alone was associated with more severe, and playing with another child less severe, internalising problems.

Step 3, with the addition of physical activity and contact with nature variables, was significant improvement of model fit from Step 2; Δ*χ* ^2^ (6) = 24.78, *p* < .001. Both the main effects of play variables from Step 2 retained their significance and pattern (Playing alone: beta = 0.28 (SE = 0.10), *t* (2235) = 2.75, *p* = 0.006, and playing with another child: beta = −0.50 (SE = 0.13), *t* (1972) = −3.73, *p* < .001). Furthermore, physical activity had a significant negative main effect on internalising problems (beta = −0.50 (SE = 0.13), *t* (2007) = −3.75, *p* < .001). There was a significant statistical interaction between linear time and physical activity (beta = −0.54 (SE = 0.25), *t* (1741) = 2.15, *p* = 0.032); specifically, parents reported a slight decrease in internalising problems over time among preschoolers who spent more time physically active, whilst parents reported a slight increase over time in internalising problems in children who were less active (Figure 1D).

#### Externalising problems

The results for all three steps are shown in full in Table 3. These models explained around 76% of variance in children's externalizing problems with 5% of variance explained by fixed effects only (see Supporting Information S1: Table S3 for full model fit statistics). This time, adding play variables and their interactions with time in Step 2 did not significantly improve the model fit from Step 1; *χ* ^2^ (9) = 11.36, *p* = 0.252. However, the addition of physical activity and contact with nature variables in Step 3 was significant improvement of model fit from Step 2; Δ*χ* ^2^ (6) = 17.74, *p* = 0.007. There was a significant negative main effect of physical activity on preschooler's externalising problems (beta = −0.33 (SE = 0.16), *t* (1908) = −2.10, *p* = 0.036). Furthermore, there was a significant statistical interaction between linear time and physical activity (beta = −0.84 (SE = 0.30), *t* (1691) = −2.78, *p* = 0.006). Similarly to the previous analyses on where children played, parents reported a decrease in externalising problems among preschoolers who spent more than 30 min per day being physically active, whilst for less active children symptoms were more stable over time (Figure 1B). Conversely, the statistically significant interaction between linear time and contact with nature (beta = 0.54 (SE = 0.27), *t* (1699) = 1.98, *p* = 0.048) meant that, parents reported a stronger decrease in externalising problems over time among preschoolers who spent less than 30 min per day in contact with nature than those who spent more than 30 min per day in contact with nature, although those spending less time in contact with nature had higher symptom levels throughout (Figure 1C).

## DISCUSSION

This study examined how two specific components of preschoolers play, *where* and *with whom* they played, were related to their mental health (internalising and externalising problem severity) during the early Covid‐19 restrictions. Whilst previous research has shown broad associations between children's play and mental health, there is a lack of longitudinal research and it is unclear whether play in certain contexts might be particularly important for young children's mental health.

We hypothesised that time spent playing outside and with others would be associated with better mental health in preschoolers. Our findings provide some support for these hypotheses. Time spent playing inside and outside was associated with lower internalising problems across time, with a larger effect found for outside play. For externalising problems, more inside play was associated with higher externalising problems, whereas more outside play was associated with fewer problems. Furthermore, we found an interaction between inside play and linear time, showing children who spent less time playing indoors, over time, showed a declining (improving) trajectory of externalising problems, whereas those who spent more time playing indoors over time maintained their level of externalising problems over time. Taken together the results show that outdoor play is associated with better mental health in preschool‐aged children, whereas indoor play shows inconsistent associations across internalising and externalising psychopathology. Importantly, these associations hold after controlling for physical activity levels and contact with nature.

These results support, in part, previous cross‐sectional research with older children. For example, Dodd et al. (2023) found that more time spent playing outdoors was associated with lower internalising problems, whereas more time spent playing indoors was not. Whilst the direct comparison between indoor and outdoor play is rarely made, further support for our findings comes from literature focused on the benefits of outdoor play specifically. For example, Piccininni et al. (2018) found that more outdoor play was associated with lower psychosomatic symptoms in adolescent girls; Pérez‐del‐Pulgar et al. (2021) reported that residential proximity to outdoor play spaces was associated with a lower prevalence of psychological disorders in pre‐adolescents, and Flouri et al. (2014) found associations between using parks and playgrounds and both internalising and externalising symptoms. The majority of previous research is cross‐sectional and focused on older children and adolescents. The current study therefore extends the existing literature by demonstrating that in preschool children, more outdoor play during Covid‐19 restrictions was associated with lower internalising and externalising problems over time, even after controlling for physical activity and contact with nature.

Our findings also show that *with whom* preschoolers played was related to their internalising problems. Specifically, more time playing with other children was linked to less severe internalising problems across time, whereas more time spent playing alone was associated with more severe internalising problems. No direct associations with externalising problems were found for time spent playing alone nor for time spent playing with other children. Whilst an interaction between time spent playing alone and linear time was found for externalising problems, this did not hold once covariates were included. Time spent playing with parents was not significantly associated with preschoolers' mental health. These results highlight that play with other children may be important for supporting good mental health during early childhood, in particular preventing internalising problems such as anxiety and low mood. This effect might be due to the key role that play with other children has in helping to prevent loneliness and social isolation, both of which are linked to higher rates of emotional difficulties in young children (Matthews et al., 2016). Importantly, this does not mean that solitary play is necessarily negative. Indeed, theory and research suggest that solitary play offers a range of developmental benefits, including creativity (Sumaroka & Bornstein, 2008) and language development (Zhao & Gibson, 2022); as Coplan and colleagues outline, solitary activities are heterogenous and can have both costs and benefits (Coplan et al., 2021). These data were collected during the Covid‐19 pandemic‐related restrictions, when children were particularly isolated, and where solitary play was to some extent enforced rather than freely chosen by children. Our interpretation of these findings is therefore that being able to play with other children is beneficial and not having adequate opportunity for this social play may a negative impact on children's internalising symptoms. We do not interpret the data as indicating that solitary play has a negative effect on children's mental health. It is also worthwhile considering that, even with a longitudinal design, we cannot be certain about causation and it is possible that children who have poorer mental health over time make different choices about how they play, or that unmeasured confounds explain the associations found. Only experimental designs can provide clear evidence of causality.

Whilst not the primary focus of the present research, we included estimations of physical activity and contact with nature as covariates given that some of the effects of play may be due to increased physical activity, rather than play per se. All of the main effects of play held when physical activity and contact with nature were included in the models, which demonstrates that the above findings are unlikely to be attributable to these potential confounding factors. Parent education level and parent mental health symptoms were also controlled for in all models. Physical activity was found to be significantly associated with internalising and externalising problems and to interact with time such that when preschool‐aged children spent more time physically active over time, their internalising and externalising problems decreased more over time. These effects were clearer in the ‘with whom’ models than the models that included inside and outside play, likely due to associations between outdoor play and physical activity. The findings regarding physical activity are consistent with physical activity guidelines that suggest being more physically active is associated with better mental health (UK Government Department of Health and Social Care, 2019) and with other research undertaken during the pandemic; for example, Wright et al. (2021) found that physical activity is a strong predictor of better mental health in children in the UK.

In general, contact with nature was not closely associated with internalising or externalising problems within our sample, at least after controlling for demographic, play and physical activity variables. For externalising problems there was a significant interaction that indicated that children who spent *less* time in nature over time showed greater decline (improvement) in externalising problems over time. This is in contradiction to substantial evidence showing that time in nature is beneficial for mental health in children and young people (see Tillman et al., 2018 for a review). Nevertheless, Figure 1C shows that the children who were spending less time in nature had higher externalising problems at baseline and, over time, these declined to a level consistent with those who were spending more time in nature at baseline. Thus, across time, preschool children spending more time in nature had fewer, or equivalent, externalising problems, relative to those who spent less time in nature, which is more consistent with previous findings. Importantly, contact with nature was only examined as a covariate, not as a direct effect, and the measurement of this construct is relatively crude, so these secondary findings should be treated with caution.

It is noteworthy that, whilst statistically significant, the fixed effects only accounted for between 4% and 6% of the variance in children's internalising and externalising problems. This indicates that play, and physical activity/contact with nature, are associated with mental health in young children but that they play a relatively minor role. Clearly a wide range of other factors affect preschooler's mental health, which are not measured in this study, so this is perhaps not surprising. Importantly though, at a population level small effects can be meaningful (Matthay et al., 2021), and play is something that children enjoy doing and can be provided for free or at very low cost, so it remains an important target for policy and intervention.

### Limitations

The results should be considered in light of the study's limitations. First, the generalisability of our findings is constrained by the specific context in which we collected data (i.e., during the Covid‐19 lockdown), which makes it unclear if the associations detected would be present in more typical circumstances. Further, this context meant that the longitudinal timeframe was relatively short and that the nature of outdoor play may have been somewhat atypical due to playground closures and social distancing measures. Importantly though, using data collected during lockdown also represents a strength of the study because young children's mental health and wellbeing can change rapidly, especially during periods of challenge, and many of children's usual activities were restricted, allowing for a clearer examination of how play might be associated with mental health. Second, we are unable to generalise findings to the wider UK population because the sample was heavily weighted with families from relatively affluent, White British backgrounds and which under‐represented families from low‐income households and households of other ethnic minorities. To improve diversity and generalizability, future research recruitment should be targeted towards minority populations, for example, translating recruitment advertisements into other languages, actively working with minority groups through faith and community associations, and using targeted social media campaigns. Third, we used a relatively simple, parent‐report measure of play, physical activity and contact with nature. In large‐scale longitudinal surveys it is often necessary to keep questions short but we recognise that this is a limitation for a number of reasons. For example, the distribution of responses across the ordinal categories for the children's activities variables meant that we had to dichotomise these variables to retain statistical power and produce reliable estimates of effect. This means that we are not able to make conclusions about how the ‘dosage’ of play is associated with outcomes. Further, no definition of play was given to survey respondents. Whilst research shows that adults are quite consistent with one another at recognising play (Smith & Vollstedt, 1985), a definition would have helped improve rigour and would have provided clarity for respondents about whether digital play should be included as play or not. Without this clarity, it is unclear whether indoor and solitary play in particular might be confounded with screen‐based play. These would be useful questions to explore in future work where ideally, parent‐report would be complemented by objective or multi‐informant data.

### Future research

Whilst this research provides some insight into associations between play and mental health in young children, there is substantial scope for future research to examine long‐term impacts of specific early play experiences, and the mechanisms through which play might affect children and young people's mental health. This research will not be straightforward because defining and measuring play is a challenge (FitzGibbon et al., 2024); play is so ubiquitous across childhood it is complex to capture children's rich, diverse play experiences. There are also likely developmental effects, where certain types of play may be particularly important at certain ages. Despite these challenges, this is important research given the context of declining outdoor play opportunities for children (Clements, 2004; Dodd et al., 2021). More detailed measurement of children's play within cohort studies would be invaluable, and natural experiments with outcomes assessed where new play spaces are provided, could also help to move the field forward and address some of these unanswered questions. It may also be valuable for future research to explore how sibling presence and play opportunities might interact to influence children's mental health and wellbeing. We did not include the presence of siblings as a covariate in our analyses because there is a lack of theoretical and empirical support for an association between the presence of siblings and mental health in young children. However, sibling play may affect sibling relationship quality, which is associated with mental health. It may therefore be interesting to explore how and when play can support positive sibling relationships.

## CONCLUSION

In conclusion, our findings show that play with other children and outdoor play appear to be particularly important for good mental health in young children. Furthermore, the importance of children's play was unaffected by their activity levels and contact with nature. Future longitudinal studies should objectively assess young children's play to further clarify its role in protecting children's mental health.

## AUTHOR CONTRIBUTIONS

**Helen F. Dodd:** Conceptualization; funding acquisition; methodology; project administration; writing—original draft; writing—review and editing. **Ella Patterson:** Conceptualization; data curation; investigation; methodology; project administration; writing—review and editing. **Simona Skripkauskaite:** Conceptualization; data curation; formal analysis; visualization; writing—review and editing. **Peter J. Lawrence:** Conceptualization; data curation; funding acquisition; methodology; project administration; supervision; writing—original draft; writing—review and editing.

## CONFLICT OF INTEREST STATEMENT

HD is an unpaid trustee of Play England and has done paid consultancy work for Play Wales, Play Scotland, Red Consulting, IKEA. None of these organisations had any role in the funding or conduct of this research. The remaining authors have declared that they have no competing or potential conflicts of interest.

## ETHICAL CONSIDERATIONS

Ethical approval for the study was granted by The University of Southampton Research Ethics Committee: ERGO 56217 on 16th April, 2020. Parents/carers provided informed consent.

## Supporting information

Supporting Information S1

## Data Availability

Data available on request from the authors who are in the process of making the data open access via the UK Data Service.
