# Supplementary material for: Longitudinal associations between play experiences and trajectories of preschoolers' mental health from April–July, 2020
Source: JCPP Adv. 2026 Feb 10;6(3):e70076. doi: 10.1002/jcv2.70076 (PMC13339685; doi:10.1002/jcv2.70076)
Supplement: Supplementary file 1 [file JCV2-6-e70076-s001.docx]

# Longitudinal associations between play experiences and trajectories of preschoolers’ mental health from April – July, 2020.

**Supporting Information**

**Table S1**

Response frequency for play variables (note responses given on 5 point scale)

|  | **1 (N=454)** | **2 (N=789)** | **3 (N=713)** | **4 (N=574)** | **Overall (N=2530)** |
| --- | --- | --- | --- | --- | --- |
| **Play indoors** |  |  |  |  |  |
| 0 | 0 (0%) | 0 (0%) | 1 (0.1%) | 1 (0.2%) | 2 (0.1%) |
| 1 | 2 (0.4%) | 5 (0.6%) | 4 (0.6%) | 3 (0.5%) | 14 (0.6%) |
| 2 | 106 (23.3%) | 140 (17.7%) | 148 (20.8%) | 148 (25.8%) | 542 (21.4%) |
| 3 | 249 (54.8%) | 410 (52.0%) | 395 (55.4%) | 293 (51.0%) | 1347 (53.2%) |
| 4 | 97 (21.4%) | 234 (29.7%) | 165 (23.1%) | 129 (22.5%) | 625 (24.7%) |
| **Play outdoors** |  |  |  |  |  |
| 0 | 9 (2.0%) | 14 (1.8%) | 6 (0.8%) | 4 (0.7%) | 33 (1.3%) |
| 1 | 23 (5.1%) | 56 (7.1%) | 48 (6.7%) | 30 (5.2%) | 157 (6.2%) |
| 2 | 237 (52.2%) | 435 (55.1%) | 411 (57.6%) | 312 (54.4%) | 1395 (55.1%) |
| 3 | 152 (33.5%) | 217 (27.5%) | 193 (27.1%) | 189 (32.9%) | 751 (29.7%) |
| 4 | 33 (7.3%) | 67 (8.5%) | 55 (7.7%) | 39 (6.8%) | 194 (7.7%) |
| **Play alone** |  |  |  |  |  |
| 0 | 14 (3.1%) | 24 (3.0%) | 19 (2.7%) | 20 (3.5%) | 77 (3.0%) |
| 1 | 133 (29.3%) | 213 (27.0%) | 193 (27.1%) | 164 (28.6%) | 703 (27.8%) |
| 2 | 247 (54.4%) | 404 (51.2%) | 368 (51.6%) | 292 (50.9%) | 1311 (51.8%) |
| 3 | 47 (10.4%) | 104 (13.2%) | 102 (14.3%) | 80 (13.9%) | 333 (13.2%) |
| 4 | 13 (2.9%) | 44 (5.6%) | 31 (4.3%) | 18 (3.1%) | 106 (4.2%) |
| **Play with a parent** |  |  |  |  |  |
| 0 | 1 (0.2%) | 0 (0%) | 1 (0.1%) | 0 (0%) | 2 (0.1%) |
| 1 | 14 (3.1%) | 34 (4.3%) | 48 (6.7%) | 52 (9.1%) | 148 (5.8%) |
| 2 | 164 (36.1%) | 340 (43.1%) | 347 (48.7%) | 304 (53.0%) | 1155 (45.7%) |
| 3 | 189 (41.6%) | 284 (36.0%) | 220 (30.9%) | 160 (27.9%) | 853 (33.7%) |
| 4 | 86 (18.9%) | 131 (16.6%) | 97 (13.6%) | 58 (10.1%) | 372 (14.7%) |
| **Play with a child** |  |  |  |  |  |
| 0 | 219 (48.2%) | 374 (47.4%) | 339 (47.5%) | 256 (44.6%) | 1188 (47.0%) |
| 1 | 25 (5.5%) | 65 (8.2%) | 67 (9.4%) | 58 (10.1%) | 215 (8.5%) |
| 2 | 88 (19.4%) | 141 (17.9%) | 129 (18.1%) | 119 (20.7%) | 477 (18.9%) |
| 3 | 73 (16.1%) | 112 (14.2%) | 115 (16.1%) | 84 (14.6%) | 384 (15.2%) |
| 4 | 49 (10.8%) | 97 (12.3%) | 63 (8.8%) | 57 (9.9%) | 266 (10.5%) |
| **Physical activity** |  |  |  |  |  |
| 0 | 4 (0.9%) | 13 (1.6%) | 6 (0.8%) | 2 (0.3%) | 25 (1.0%) |
| 1 | 55 (12.1%) | 113 (14.3%) | 100 (14.0%) | 64 (11.1%) | 332 (13.1%) |
| 2 | 296 (65.2%) | 479 (60.7%) | 424 (59.5%) | 332 (57.8%) | 1531 (60.5%) |
| 3 | 83 (18.3%) | 142 (18.0%) | 141 (19.8%) | 151 (26.3%) | 517 (20.4%) |
| 4 | 16 (3.5%) | 42 (5.3%) | 42 (5.9%) | 25 (4.4%) | 125 (4.9%) |
| **Contact with nature** |  |  |  |  |  |
| 0 | 12 (2.6%) | 21 (2.7%) | 13 (1.8%) | 8 (1.4%) | 54 (2.1%) |
| 1 | 61 (13.4%) | 119 (15.1%) | 106 (14.9%) | 86 (15.0%) | 372 (14.7%) |
| 2 | 270 (59.5%) | 454 (57.5%) | 415 (58.2%) | 318 (55.4%) | 1457 (57.6%) |
| 3 | 88 (19.4%) | 151 (19.1%) | 142 (19.9%) | 132 (23.0%) | 513 (20.3%) |
| 4 | 23 (5.1%) | 44 (5.6%) | 37 (5.2%) | 30 (5.2%) | 134 (5.3%) |

**Table S2**

Model fit indices for internalizing problem models

|  |  | Play Where | | | | | | |  | Play with Whom | | | | | | |
| --- | --- | --- | --- | --- | --- | --- | --- | --- | --- | --- | --- | --- | --- | --- | --- | --- |
|  |  | *df* | *AIC* | *BIC* | ∆*χ^2^* | *p* | Marginal *R^2^* | Conditional *R^2^* |  | *df* | *AIC* | *BIC* | ∆*χ^2^* | *p* | Marginal *R^2^* | Conditional *R^2^* |
| Step 0 | | |  |  |  |  |  |  |  |  |  |  |  |  |  |  |
|  | Quadratic growth model | | | | | |  |  |  |  |  |  |  |  |  |  |
|  |  | 5 | 11394 | 11423 |  |  | .004 | .722 |  | 5 | 11394 | 11423 |  | .004 | .722 |  |
| Step 1 | | |  |  |  |  |  |  |  |  |  |  |  |  |  |  |
|  | + Control variables | | | | | |  |  |  |  |  |  |  |  |  |  |
|  |  | 7 | 11310 | 11351 | 87.66 | <.001 | .037 | .705 |  | 7 | 11310 | 11351 | 87.66 | <.001 | .037 | .705 |
| Step 2 | | |  |  |  |  |  |  |  |  |  |  |  |  |  |  |
|  | + Play behaviours * Time | | | | | |  |  |  |  |  |  |  |  |  |  |
|  |  | 13 | 11294 | 11370 | 27.34 | <.001 | .045 | .704 |  | 16 | 11300 | 11393 | 28.09 | <.001 | .050 | .704 |
| Step 3 | | |  |  |  |  |  |  |  |  |  |  |  |  |  |  |
|  | + Other activities * Time | | | | | |  |  |  |  |  |  |  |  |  |  |
|  |  | 19 | 11291 | 11402 | 15.55 | .016 | .049 | .705 |  | 22 | 11287 | 11415 | 24.78 | <.001 | .056 | .704 |

*Note*. Step 0 and Step 1 were the same for ‘Play Where’ and ‘Play with Whom’ models.

**Table S3**

Model fit indices for externalizing problem models

|  |  | Play Where | | | | | | |  | Play with Whom | | | | | | |
| --- | --- | --- | --- | --- | --- | --- | --- | --- | --- | --- | --- | --- | --- | --- | --- | --- |
|  |  | *df* | *AIC* | *BIC* | ∆*χ^2^* | *p* | Marginal *R^2^* | Conditional *R^2^* |  | *df* | *AIC* | *BIC* | ∆*χ^2^* | *p* | Marginal *R^2^* | Conditional *R^2^* |
| Step 0 | | |  |  |  |  |  |  |  |  |  |  |  |  |  |  |
|  | Quadratic growth model | | | | | |  |  |  |  |  |  |  |  |  |  |
|  |  | 5 | 12499 | 12528 |  |  | .007 | .770 |  | 5 | 12499 | 12528 |  |  | .007 | .770 |
| Step 1 | | |  |  |  |  |  |  |  |  |  |  |  |  |  |  |
|  | + Control variables | | | | | |  |  |  |  |  |  |  |  |  |  |
|  |  | 7 | 12400 | 12441 | 102.99 | <.001 | .046 | .758 |  | 7 | 12400 | 12441 | 102.99 | <.001 | .046 | .758 |
| Step 2 | | |  |  |  |  |  |  |  |  |  |  |  |  |  |  |
|  | + Play behaviours * Time | | | | | |  |  |  |  |  |  |  |  |  |  |
|  |  | 13 | 12394 | 12470 | 17.50 | .008 | .050 | .759 |  | 16 | 12406 | 12500 | 11.36 | .252 | .048 | .759 |
| Step 3 | | |  |  |  |  |  |  |  |  |  |  |  |  |  |  |
|  | + Other activities * Time | | | | | |  |  |  |  |  |  |  |  |  |  |
|  |  | 19 | 12392 | 12503 | 14.24 | .027 | .053 | .760 |  | 22 | 12401 | 12529 | 17.74 | .007 | .052 | .760 |

*Note*. Step 0 and Step 1 were the same for ‘Play Where’ and ‘Play with Whom’ models
